# Haplotypes in the promoter region of the *CIDEC* gene associated with growth traits in Nanyang cattle

**DOI:** 10.1038/srep12075

**Published:** 2015-07-20

**Authors:** Jing Wang, Liu-shuai Hua, Hong Pan, Liang-zhi Zhang, Ming-xun Li, Yong-zhen Huang, Zhuan-jian Li, Xian-yong Lan, Chu-zhao Lei, Cong-jun Li, Hong Chen

**Affiliations:** 1 College of Animal Science and Technology, Northwest A&F University, Shaanxi Key Laboratory of Molecular Biology for Agriculture, Yangling Shaanxi 712100, People’s Republic of China; 2 Institute of Animal Husbandry and Veterinary Science, Henan Academy of Agricultural Sciences, Zhengzhou Henan, 450002, People’s Republic of China; 3 United States Department of Agriculture-Agricultural Research Service, Bovine Functional Genomics Laboratory, Beltsville, Maryland 20705, United States of America

## Abstract

Cell death-inducing DFFA-like effector c (CIDEC, also known as *Fsp27*) has emerged as an important regulator of metabolism associated with lipodystrophy, diabetes, and hepatic steatosis. It is required for unilocular lipid droplet formation and optimal energy storage. The mechanism between this gene and livestock growth traits, however, has yet to be reported. In this study, we found ten novel single nucleotide polymorphisms (SNPs) in the 5’ transcriptional region of *CIDEC* in Nanyang (NY) cattle, which are located in the recognition sequences (potential *cis*-acting elements) of 22 transcription factors, and the nine haplotypes represent nine different combinations of polymorphic potential *cis*-acting elements. The results indicated that individuals with the H8-H8 diplotype had heavier body weights and faster growth rates (*P* < 0.01) at 18th months than those with H1-H8. We evaluated the transcriptional activities of different haplotypes *in vitro*, the results were consistent with the association analysis. The H8 haplotype had 1.88-fold (*P* < 0.001) higher transcriptional activity than the H1 haplotype. We speculate that the haplotypes of the potential *cis*-acting elements may affect the transcriptional activity of *CIDEC*, thus affecting the growth traits of cattle. This information may be used in molecular marker-assisted selection of cattle breeding in the future.

The family of CIDE proteins, comprising three members (CIDEA, CIDEB, and CIDEC), have emerged as important regulators in various aspects of metabolism^1^. *CIDEC*, also named fat-specific protein 27 (*Fsp27*) in rodents and *CIDE3* in humans, was recognized to be vital in unilocular lipid droplet formation and optimal energy storage. *CIDEC*-knockout mice have a lean phenotype and atrophic adipose tissue as a result of high energy expenditure. This mouse line is also protected against diet-induced obesity and insulin resistance^2^,^3^. In human foam cells, *CIDEB* and *CIDEC* were shown to be important for the formation and catabolism of intracellular lipid droplets and the process of atherosclerosis^4^. *CIDEC* was also found to be expressed in the steatotic liver of the type-II diabetes model mice. The physiological roles of *CIDEC* in adipose tissue and the liver demonstrate its significance as a factor in the development of metabolic disorders^5^.

Variations in *CIDEC* are associated with abnormal lipid metabolism. For example, a female patient who was found to be homozygous for a premature truncation variation in the conserved CIDE-C-terminal domain had partial lipodystrophy and insulin-resistant diabetes^6^. Based on previous studies, we hypothesized that the *CIDEC* gene plays an important role in bovine growth traits; however, to date there is no research on the variations in the bovine *CIDEC* gene. In this study, we found nine potential *cis*-acting element haplotypes involving ten novel variations in the bovine *CIDEC* gene, and then we assessed the genetic impacts of these haplotypes on bovine body weight (BW) and average daily gain (ADG). In addition, we analyzed the mechanisms of these genetic impacts based on transcriptional activity. The results of this research will contribute to the understanding of the regulatory mechanisms of the *CIDEC* gene, and may be used in molecular marker-assisted selection of live cattle breeding in the future.

## Results

### Ten novel SNPs were identified in the transcriptional regulatory region of the *CIDEC* gene

**Figure 1:**
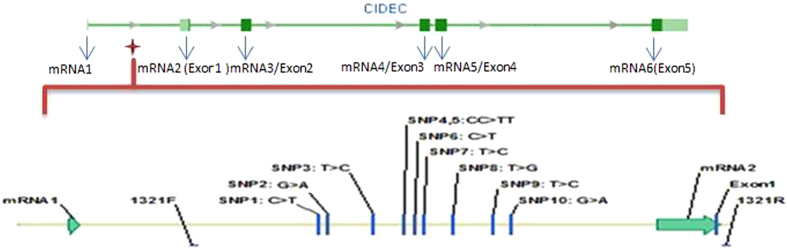
The structure of bovine *CIDEC* and the SNPs in intron 1 and its flanking region. Note: 1321F and 1321R were the primers used to detect the genotypes.

The bovine *CIDEC* gene is located on chromosome 22 and encodes 222 amino acids, including five exons. In the present study, polymorphisms in the 5’ region of the bovine *CIDEC* gene were identified by DNA pool sequencing, and ten novel SNPs were identified [AC_000179.1: *g.−501 G* *>* *A; −546 T* *>* *C; −643 T* *>* *G; −714 T* *>* *C; −727 C* > *T; −762 C* > *T; −763 C* > *T; −841 T* > *C; −956 G* > *A; −974 C* > *T*]. These ten SNPs were novel and have been deposited in the GenBank database (see Fig. 1 and Supplementary Table S1).

As shown in Supplementary Table S2, the genotype and allele frequencies and the diversity parameters of the bovine *CIDEC* were directly calculated for all 213 animals. There were three genotypes for each locus, except g.−714 T > C, for which the CC genotype was not observed. The distribution of the genotype and allele frequencies of g.−956 G > A; −841 T > C; −763 C > T; −727 C > T, and −546 T > C were similar, and g.−643 T > G and −501 G > A had the same genotype frequency distribution.

The values of *He* approached 0.500, and the g.−714 T > C locus had the lowest *He* value (0.025). The values of *Ne* approached 2.000 for most of loci except the g.−714 T > C locus, which had a value of 1.024. Apart from g.−714 T > C, the other loci had intermediate polymorphisms.

### SNP variation in potential *cis*-acting elements of the *CIDEC* gene

A search of the Genomatix database (http://www.genomatix.de) revealed 208 potential *cis*-acting elements in the 1.321 kb region (5’ relative to the translation start site, including these ten SNPs) of *CIDEC*. Therefore, those recognition sequences containing one or two SNPs, or being adjacent to SNPs, were evaluated. Accordingly, similarity to a total of 25 known regulatory motifs was scored on both strands of the bovine *CIDEC* sequence, of which 16 were located in the plus strand and nine in the minus strand. Detailed information on the SNPs and motifs is presented in Table 1.

### Haplotypes of the potential *cis*-acting elements in the *CIDEC*

The degrees of linkage disequilibrium between these ten *CIDEC* SNPs are shown in Supplementary Table S3. The *D’* values ranged from 0.895 to 1.000, and the *r*^*2*^ values ranged from 0.003 to 1.000. It is noteworthy that two linkage disequilibrium blocks were observed among these SNPs (block 1: g. −956 G > A, g. −841 T > C, and g. −763 C > T, *r*^*2*^ = 1.000; block 2: g. −643 T > G and g. −501 G > A, *r*^*2*^ = 1.000).

Haplotypes were reconstructed with the PHASE computer program. Nine haplotypes were identified in this sample of NY cattle (Table 2). The frequencies of the haplotypes ranged from 0.000 to 0.464. H8 had the highest frequency (0.464). Combined with the information from the potential *cis*-acting elements in Table 1, we found that the potential *cis*-acting elements were changed in all nine haplotypes. H1 and H2 both led to fourteen mutated potential *cis*-acting element sequences (of which four potential *cis*-acting elements had variations in their core sequence), followed by H5, which led to nine mutated *cis*-acting element sequences. H8, which resulted in only three variations in potential *cis*-acting elements, had the highest frequency among the NY cattle studied. We found that this bovine population was mainly composed of individuals with fewer variations in potential *cis*-acting elements.

### Haplotypes in the promoter region of the *CIDEC* were associated with the BW and ADG of NY cattle

To further assess the associations between the diplotypes of these ten SNPs and both BW and ADG in NY cattle, the diplotypes of the ten SNPs were constructed. As shown in Table 2, there were nine haplotypes in the NY breed. Because the frequencies of H3 (0.024), H5 (0.012), H6 (0.000), H7 (0.012), and H9 (0.012) were small, our association analysis for the effect of diplotypes excluded the diplotypes related to these haplotypes. Therefore, four haplotypes were included: H1 (0.119), H2 (0.155), H4 (0.202), and H8 (0.484), and five diplotypes were used in the correlation analysis (Table 3). Individuals with the H8-H8 (TT-GG-TT-CC-CC-CC-TT-GG-TT-GG) diplotype had heavier BW and higher ADG at the 18th months, as compared to individuals with all other diplotypes (*P* < 0.05) (Table 3). However, the associations between the diplotypes and BW and ADG were not significant at other time points (*P* > 0.05), so the detailed results are not displayed in this paper.

### Haplotypes of the potential *cis*-acting elements in the *CIDEC* possessed different transcriptional activities

**Figure 2:**
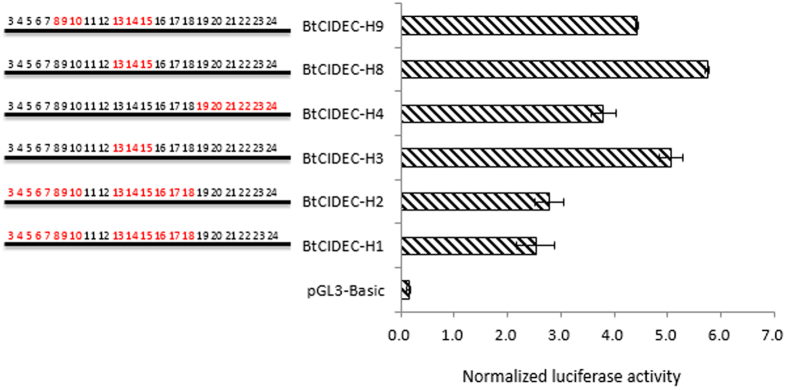
*In vitro* constitutive activities of the bovine *CIDEC* haplotypes. The numbers in the left part of the figure refers to the potential *cis*-acting elements (Table 1), and the red color of the numbers indicates that there are mutations existing in the corresponding element. The histograms in the right part of the figure refer the transcriptional activities of the candidate haplotypes. The ratio of firefly luciferase to renilla luciferase was used to normalize the promoter activity. The error bars represent the standard error of the mean (SE) from three independent assays.

Pooled genomic DNA from 213 NY cattle was used as a template, and a pair of specific primers (see Supplementary Table S4) were used to get these different haplotypes (see Table 2) by polymerase chain reaction (PCR), and then the transcriptional activities of these haplotypes were determined using a dual-luciferase reporter assay system in a mouse embryonic fibroblast-adipose-like cell line (3T3-L1). The H8 haplotype had a 2.27-fold (*P* < 0.001) higher activity comparing with the H1 haplotype (Fig. 2); these two haplotypes differed at 11 potential *cis*-acting elements (3–10 and 16–18 in Table 1).

## Discussion

In leptin-deficient (*ob/ob*) mice, the *Fsp27* expression level was significantly up-regulated in the white adipose tissue (WAT) and liver^7^. In the WAT of *Fsp27*-deficient mice, the expression levels of BAT-selective genes and WAT-selective genes were significantly up-regulated and down-regulated, respectively, the WAT was “browning”^8^. *Fsp27*^*-/-*^ mice had lower visceral fat volume, smaller adipocyte size of WAT and brown adipose tissue (BAT)^9^, higher glucose intake rates, higher lipolysis rates, dramatically lower levels of TAG, and increased mitochondrial volumes in white adipocytes^10^. *Fsp27/leptin* double-deficient mice were resistant to diet-induced obesity and displayed increased insulin sensitivity^8^,^10^. Overall, animals with *Fsp27* deficiencies have lean phenotypes with higher energy expenditure.

*CIDEC* is mainly expressed in human subcutaneous adipocytes that are located on the surface of lipid droplets; its expression increases during differentiation^11^ and declines in response to reduced caloric intake^3^. In obese humans, the hepatic expression of *CIDEC* increases and its expression is strongly correlated to BMI at the time of gastric bypass surgery^12^. In BMI-matched obese humans, *CIDEC* levels in WAT are positively correlated with insulin sensitivity. In human adipocytes, insulin may increase *CIDEC* mRNA expression, which suggests that it plays a role in controlling adipose lipolysis and thus circulating fatty acids^13^,^14^. Overall, *CIDEC* gene is a novel regulator of obesity, type 2 diabetes, and liver steatosis.

In this study, the cattle with the H8-H8 diplotype showed heavier BW and higher ADG than individuals with other diplotypes at 18th months (*P* = 0.016 and *P* = 0.011, respectively). Why did individuals with different diplotypes show different growth traits? We hypothesize that the variations in the flanking region of the *CIDEC* may change the associated *cis*-acting elements and thus its transcription^15^,^16^,^17^. In the current study, these nine haplotypes represent nine potential *cis*-acting element compositions, and the dual-luciferase reporter assay indicated that the transcriptional activities was associated with these mutations: the individuals with fewer variations in potential *cis*-acting elements had higher transcriptional activity. For example, the BtCIDEC-H8 with the least mutated potential *cis*-acting elements (only three) had the highest transcriptional activity, and individuals with the H8-H8 diplotype had the highest BWs. These results are consistent with previous research on rodents and human in that individuals with higher *CIDEC* expression levels had better growth rates.

It is worth mentioning that the associations between diplotypes with BW and ADG were significant only at 18 months-old; there was no correlation at other time points, which may be explained by an inconsistent bovine growth rate^18^. Further determination of the expression profile of *CIDEC* during bovine growth and development may help us to understand the temporal heterogeneity of this biological process.

We detected ten novel SNPs in NY cattle *CIDEC* gene, and these SNPs led to variations in 22 potential *cis-*acting elements. Haplotypes with different combinations of potential *cis*-acting elements exhibited different transcriptional activity, and the differences in transcriptional activity may be the biological basis of the association between the potential *cis*-acting elements and growth traits. This information may be used in molecular marker-assisted selection of cattle breeding in the future.

## Methods

### Ethics statement

The experiments and animal care were performed according to the Regulations for the Administration of Affairs Concerning Experimental Animals (Ministry of Science and Technology, China, 2004) and approved by the Institutional Animal Care and Use Committee (College of Animal Science and Technology, Northwest A&F University, China). Adult cattle were allowed access to feed and water ad libitum under normal conditions and all efforts were made to minimize suffering.

### Cattle population, data collection, and genomic DNA isolation

The cattle used in this research were an indigenous Chinese breed, NY cattle. This breed is suited for large-scale farming and meat production and is distributed in the Henan province of China. Many other provinces have introduced NY cattle to improve their local cattle breeds. The test herd used in this study was reared at the Henan Nanyang Cattle Conservation Farm, where the cattle were kept under the same conditions. The animals were weaned at an average of six months age and raised on a diet of corn and corn silage.

The variable under investigation in this study were body weight (BW) in kilograms and average daily gain (ADG) in kilograms at birth (30.468 ± 2.986), six months-old (162.065 ± 19.237, 0.731 ± 0.099), 12 months-old (226.032 ± 25.766, 0.355 ± 0.102), 18 months-old (303.387 ± 32.785, 0.429 ± 0.177), and 24 months-old (395.710 ± 40.062, 0.512 ± 0.262).

Blood samples from 213 female NY cattle (which came from different five sires and each one has one dam) were obtained by jugular venipuncture using vacuum tubes treated with 0.25% ethylene diamine tetraacetic acid (EDTA). Genomic DNA was extracted from the blood samples using a standard method^19^.

### Primer design, PCR amplification, and SNP detection

Five pairs of PCR primers were designed to amplify all exons (exons 1–6) and the 5' untranslated region (UTR) of the bovine *CIDEC* (AC_000179.1), including the intron–exon boundaries and proximal flanking regions. The primer sequences are displayed in Supplementary Table S4.

PCR amplifications were carried out using pooled genomic DNA from the 213 NY cattle as a template^20^. The 25 μL PCR reaction volume contained 50 ng of pooled genomic DNA, 0.5 μM of primer, 1 × buffer (including 1.5 mM MgCl_2_), 200 μM dNTPs, and 0.625 units of *Taq* DNA polymerase (MBI, Vilnius, Lithuania). The cycling protocol was 5 min at 95 °C; 34 cycles of 30 s at 94 °C, annealing for 30 s (the annealing temperature for each pair of primers is listed in Supplementary Table S4) and 72 °C for 30 s, and a final extension of 10 min at 72 °C. The PCR products were sequenced using an ABI PRISM 3730XL DNA sequencer (Sangon Biotech, Shanghai, China), and the variations in the bovine *CIDEC* were then analyzed with Vector NTI software (Invitrogen). To further analyze the ten variations in the 5’ flanking region, one pair of PCR primers was designed to amplify the transcriptional regulatory region of the bovine *CIDEC* (P6 in Supplementary Table S4). The PCR products for the 213 NY cattle were sequenced using an ABI PRISM 3730XL DNA sequencer, and the SNP information was analyzed using the SEQMAN package (DNA Star Lasergene software).

### Analysis of potential *cis*-acting elements in 1.321 kb of the bovine *CIDEC* sequence

The transcriptional regulatory region of the *CIDEC* (1.321 kb, AC_000179.1, from 16909073 to 16918356), which included these ten SNPs, was analyzed using MATINSPECTOR 8.0 (Genomatix software GmbH) on its default settings. Then, the potential *cis*-acting elements with variations located in or adjacent to their recognition sequences were selected for further analysis.

### Construction of linkage disequilibrium (LD) maps and haplotypes of the potential *cis*-acting elements in the *CIDEC*

The LD structure was constructed with HAPLOVIEW (version 3.32)^21^, which was used to calculate *D*′ and *r*^*2*^. Some research has shown that *r*^*2*^ is not as sensitive as *D*′ to allele frequencies; therefore, *r*^*2*^ was used as a pairwise measurement of LD in our analysis^22^.

Haplotypes were obtained for each animal using the PHASE (version 2.1) computer program^23^. Nine haplotypes for the *CIDEC* were detected among the 213 NY cattle. These haplotypes were combined with the potential *cis*-acting elements in Table 1; with the exception of −956 G > A and −974 C > T, all SNPs were located in 22 potential *cis-*acting elements. Different haplotypes corresponded to different sequences of the potential *cis-*acting elements. In total, there were nine haplotypes that represented nine haplotypes of potential *cis-*acting elements.

### Plasmid construction

Because the frequencies of the haplotypes H5, H6, and H7 (0.012, 0.000, and 0.012, respectively) were too low, haplotypes H1–H4 and H8–H9 of potential *cis*-acting elements in the *CIDEC* were amplified and inserted into the pGL3-Basic vector to determine their transcriptional activities using a dual-luciferase reporter assay system (Promega, Heidelberg, Germany). *Kpn*I and *Xho*I restriction sites were added to the forward and reverse primers, respectively. The PCR products were cleaned using a TIANquick Midi Purification Kit (Tiangen, Beijing, China) following the manufacturer’s instructions, and then digested with *Kpn*I and *Xho*I. The PCR products with sticky ends were inserted into the pGL3-Basic vector, and a sample of the ligation mix was then transferred into Top 10 *E. coli* cells (Promega). Positive clones were selected based on the restriction digestion profiles of the plasmid DNA and were further confirmed by DNA sequencing.

### Determination of the transcriptional activities of different haplotypes of potential *cis*-acting elements in the *CIDEC*

3T3-L1 cells (Type Culture Collection of the Chinese Academy of Sciences, Shanghai, China) were maintained in Dulbecco’s Modified Eagle Medium (DMEM) and supplemented with 10% newborn calf serum (NBCS) and antibiotics (100 IU/mL penicillin; 100 ug/mL streptomycin) at 37 °C and 5% CO_2_ in a normal atmosphere incubator. One day before transfection, 0.5–2 × 10^5^ cells were grown overnight to 90% confluence in 250 μL of growth medium without antibiotics in 48-well plates. In each well, 385 ng of the expression construct (H1, H2, H3, H4, H8, or H9), 15 ng of pRL-TK normalizing vector, and 1 μL of Lipofectamine 2000 (Invitrogen) were incubated with 60 μL of serum-free DMEM. The pGL3-Basic vector was used as a negative control. Transfections were performed in 250 μL of serum-free DMEM for 6 h, and then the medium was replaced with 250 μL of fresh 10% NBCS DMEM. Cell lysates were collected 24 h post-transfection and used for the measurement of the relative transcriptional activity of each fragment with the Dual-Luciferase Reporter Assay System (Promega), according to the manufacturer’s protocol. The relative luciferase activities were determined using a VICTORTM X2 Multilabel Plate Reader (PerkinElmer, Inc., Waltham, MA, USA). At least three parallel experiments were performed for statistical analysis.

### Statistical analysis

The genotype and allele frequencies of the SNPs in the bovine *CIDEC* from the 213 NY cattle were determined by direct counting. Gene homozygosity (*Ho*), heterozygosity (*He*), the effective allele number (*Ne*), and the polymorphism information content (*PIC*) were evaluated by Nei’s methods^24^.

Associations between the diplotypes of the *CIDEC* and growth traits in NY cattle were analyzed using the General Linear Model (GLM) in SPSS (version 16.0). All analyses were performed in two steps: first, a full animal model was used, and second, a reduced animal model was used. The full animal model included fixed effects for marker diplotypes, birth year, season of birth (spring vs. fall), ages of the dam and sire, farm, and random effects (permanent environment, animal, and residual). The effects associated with the season of birth, age of the dam and sire, farm, and random effects were not matched in the linear model because preliminary statistical analyses indicated that these effects did not have a significant influence on trait variability in the analyzed populations (*P* > 0.05). Thus, the reduced model, which included fixed effects for age and diplotypes, was used in the final analysis. The linear model was as follows: *Y*_*ij*_* = μ + A*_*i*_* + D*_*j*_* + E*_*ij*_, where *Y*_*ij*_ is the trait measured for the *ij*th animal, *μ* is the overall population mean, *A*_*i*_ is the fixed effect due to the *i*th age, *D*_*j*_ is the fixed effect associated with the *j*th diplotype, and *E*_*ij*_ is the random error. In this model, age and marker diplotypes were considered fixed effects and the growth traits were considered the dependent variables. The results of the multiple comparisons were corrected by Bonferroni correction, and the differences were considered significant at *P* < 0.05. The transcriptional activities for the different haplotypes of potential *cis*-acting elements were expressed as the mean ± SE.

## Additional Information

**How to cite this article**: Wang, J. *et al.* Haplotypes in the promoter region of the *CIDEC* gene associated with growth traits in Nanyang cattle. *Sci. Rep.* **5**, 12075; doi: 10.1038/srep12075 (2015).

## Supplementary Material

Supplementary Information

## Figures and Tables

**Table 1:**
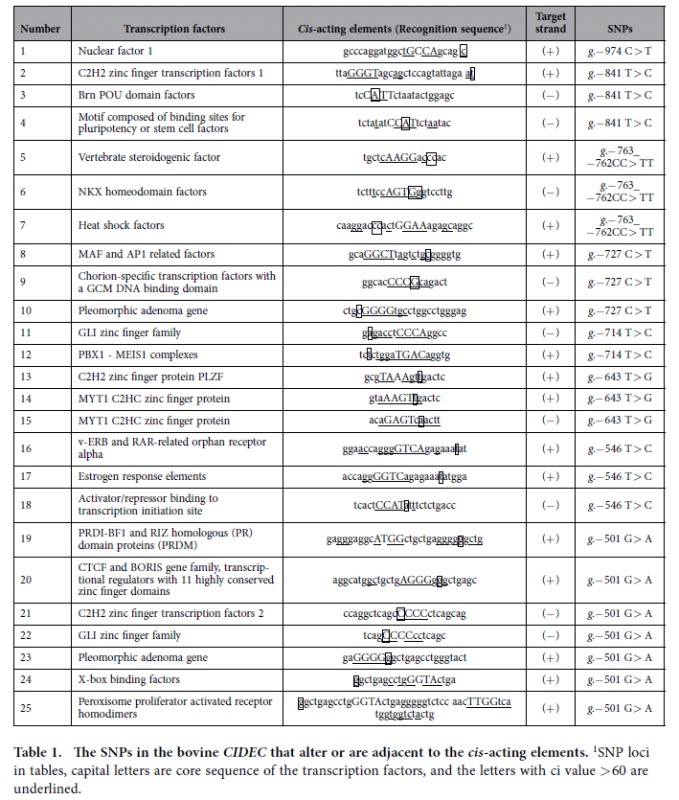
The SNPs in the bovine *CIDEC* that alter or are adjacent to the *cis*-acting elements.

^1^SNP loci in tables, capital letters are core sequence of the transcription factors, and the letters with ci value >60 are underlined.

**Table 2:** Haplotypes and haplotype frequencies of ten SNPs in bovine *CIDEC* in the NY cattle population.

**Haplotypes**	**g.−974**	**g.−956**	**g.−841**	**g.−763**	**g.−762**	**g.−727**	**g.−714**	**g.−643**	**g.−546**	**g.−501**	**Frequency**
**AC_000179.1**	**C > T**	**G > A**	**T > C**	**C > T**	**C > T**	**C > T**	**T > C**	**T > G**	**T > C**	**G > A**	**(%)**
H1	C	A	C	T	C	T	T	G	C	G	0.119
H2	C	A	C	T	T	T	T	G	C	G	0.155
H3	C	G	T	C	C	C	T	G	T	G	0.024
H4	C	G	T	C	C	C	T	T	T	A	0.202
H5	C	G	T	C	C	T	T	G	C	G	0.012
H6	C	G	T	C	T	C	T	G	T	G	0.000
H7	T	G	T	C	C	C	C	G	T	G	0.012
H8	T	G	T	C	C	C	T	G	T	G	0.464
H9	T	G	T	C	C	T	T	G	T	G	0.012

**Table 3:** Associations between the *CIDEC* diplotypes and body weight and average daily gain in NY cattle.

**Diplotype**	**Sample size**	**Growth traits (Mean ± SE)**	
**BW18**	**ADG18**		
H8-H8	62	349.000^Aa^ ± 14.056	0.680^Aa^ ± 0.075
H4-H8	40	309.667^ABb^ ± 11.477	0.460^ABb^ ± 0.061
H2-H4	28	303.200^ABb^ ± 12.572	0.341^Bb^ ± 0.067
H1-H8	34	294.000^Bb^ ± 12.572	0.449^ABb^ ± 0.067
H2-H8	34	287.727^Bb^ ± 8.476	0.352^Bb^ ± 0.045
*P* value		0.016	0.011

Values with different superscripts within the same column differ significantly at *P* < 0.05 (a, b) and *P* < 0.01 (A, B). SE: standard error. BW18: body weight at 18 months-old; ADG 18: average daily gain at 18 months-old.
