# Supplementary material for: Haplotypes in the promoter region of the CIDEC gene associated with growth traits in Nanyang cattle
Source: Sci Rep. 2015 Jul 20;5:12075. doi: 10.1038/srep12075 (PMC4648405; doi:10.1038/srep12075)
Supplement: Supplementary Information [file srep12075-s1.pdf]

Haplotypes in the promoter region of the CIDEC gene associated with growth traits in Nanyang cattle

Jing Wang[1, 2], Liu-shuai Hua [2], Hong Pan[1], Liang-zhi Zhang[1], Ming-xun Li[1], Yong-zhen Huang[1],

Zhuan-jian Li[1], Xian-yong Lan[1], Chu-zhao Lei[1] Cong-jun Li[3] and Hong Chen[1]*

Supplementary Table S1. Description of the SNPs in the bovine *CIDEC* gene.

Number	Variant type	GenBank acc. No.	DPS (nt)
1	g.-974 C>T	ss528106580	0
2	g.-956 G>A	ss528106583	20
3	g.-841 T>C	ss528106585	109
4	g.-763 C>T	ss528106587	77
5	g.-762 C>T	ss528106589	0
6	g.-727 C>T	ss528106591	24
7	g.-714 T>C	ss528106592	22
8	g.-643 T>G	ss528106594	70
9	g.-546 T>C	ss528106596	96
10	g.-501 G>A	ss528106597	44

Note: DPS, distance from previous sequence variant.

Supplementary Table S2. Genotype and allele frequencies and diversity parameters for the *CIDEC* gene in Nanyang cattle

Loci	Genotype number and frequencies			Allelic frequencies		Genetic diversity parameters			
						Ho	*He*	*Ne*	PIC
	CC	CT	TT	C	T				
g.-974 C>T	66 (0.310)	86 (0.405)	61 (0.286)	0.512	0.488	0.500	0.500	1.999	0.375
	AA	AG	GG	A	G				
g.-956 G>A	10 (0.048)	96 (0.452)	107 (0.500)	0.274	0.726	0.602	0.398	1.660	0.319
	CC	CT	TT	C	T				
g.-841 T>C	10 (0.048)	96 (0.452)	107 (0.500)	0.274	0.726	0.602	0.398	1.660	0.319
	CC	CT	TT	C	T				
g.-763 C>T	107 (0.500)	96 (0.452)	10 (0.048)	0.726	0.274	0.602	0.398	1.660	0.319
	CC	CT	TT	C	T				
g.-762 C>T	152 (0.714)	56 (0.262)	5 (0.024)	0.845	0.155	0.738	0.262	1.354	0.227
	CC	CT	TT	C	T				
g.-727 C>T	96 (0.452)	107 (0.500)	10 (0.048)	0.702	0.298	0.582	0.418	1.718	0.331
	CC	CT	TT	C	T				
g.-714 T>C	0 (0.000)	5 (0.024)	208 (0.976)	0.012	0.988	0.976	0.024	1.024	0.023
	GG	GT	TT	G	T				
g.-643 T>G	137 (0.643)	66 (0.310)	10 (0.048)	0.798	0.202	0.677	0.323	1.477	0.271
	CC	CT	TT	C	T				
g.-546 T>C	10 (0.048)	101 (0.476)	101 (0.476)	0.286	0.714	0.592	0.408	1.690	0.325
	AA	AG	GG	A	G				
g.-501 G>A	10 (0.048)	66 (0.310)	137 (0.643)	0.202	0.798	0.677	0.323	1.477	0.271

Note：*Ho*: gene homozygosity, *He*: heterozygosity, *Ne*: effective allele numbers, *PIC*: polymorphism information content.

Supplementary Table S3. The estimated values of linkage equilibrium analysis between the ten *CIDEC* SNPs in Nanyang cattle

D'	*g.*-974 C>T	*g.*-956 G>A	*g.*-841 T>C	*g.*-763 C>T	*g.*-762 C>T	*g.*-727 C>T	*g.*-714 T>C	*g.*-643 T>G	*g.*-546 T>C	*g.*-501 G>A
g.-974 C>T	-	1.000	1.000	1.000	1.000	0.895	1.000	1.000	1.000	1.000
g.-956 G>A	0.359	-	1.000	1.000	1.000	1.000	1.000	0.998	1.000	0.998
g.-841 T>C	0.359	1.000	-	1.000	1.000	1.000	1.000	0.998	1.000	0.998
g.-763 C>T	0.359	1.000	1.000	-	1.000	1.000	1.000	0.998	1.000	0.998
g.-762 C>T	0.174	0.486	0.486	0.486	-	1.000	1.000	0.954	1.000	0.954
g.-727 C>T	0.324	0.890	0.890	0.890	0.432	-	1.000	0.998	1.000	0.998
g.-714 T>C	0.013	0.005	0.005	0.005	0.002	0.005	-	1	1.000	1.000
g.-643 T>G	0.242	0.095	0.095	0.095	0.042	0.107	0.003	-	0.998	1.000
g.-546 T>C	0.381	0.943	0.943	0.943	0.458	0.944	0.005	0.101	-	0.998
g.-501 G>A	0.242	0.095	0.095	0.095	0.042	0.107	0.003	1.000	0.101	r^2

Note: The correlation coefficients *D'* and r^2 between ten SNPs were shown in the above and below diagonal of this table, respectively. The r^2 value that equals 1.000 is colored, and bigger than 0.33 is underlined.

Supplementary Table S4. Primers for PCR amplification of the bovine *CIDEC* gene

Loci	Position*	Sequence (5'-3')	AT (ºC)	SAF (bp)	Note
P1	2473-3461	AGGAGAACATTTCAGGGTG	55	989	5'flanking region
		GCAGTGGAAGTTGGGAG			(SNP detection)
P2	3480-4101	TTTCTAATCAGGGAACTAAG	65	622	Exon 1
		ACCAAAGGACTACAGGGA			(SNP detection)
P3	4230-4903	TTCTGTCACTGGCTACTTCTCA	58	674	Exon 2
		GGAGGGTGGCAGTACATTATCA			(SNP detection)
P4	7284-8223	CATCACTTACCTTATTCTCCAG	56	940	Exon 3,4
		GACAGTTAGTTCATCCCTTCCA			(SNP detection)
P5	10851-11479	TTCTTGGTGTTTTGACCCTAATGCC	59	629	Exon 5
		AAAGAACAGAAGCACAGA			(SNP detection)
P6	2466-3786	TGGGAACAGGAGAACATTTCAG	55	1321	5'flanking region
		ACAAGATGCCAAGGTGACTTACA			(DNA sequencing)

Note: AT: annealing temperature; SAF, size of amplification fragment.

*The primers were designed based on the sequence of bovine *CIDEC* gene, the GenBank number is AC_000179.1, its GeneID is 534607. To including the 5' flanking region, we including 5000bp before the translation start site, so in our sequence the position of the translation start site is 5001.

P1-P5 were used for SNP detection by DNA pool sequencing, P6 was used to detect the 10 SNPs in the 5' flanking region.
